# Donor-transmitted squamous cell carcinoma in kidney transplant recipient successfully treated with checkpoint inhibitors: a case report

**DOI:** 10.3389/fonc.2026.1770929

**Published:** 2026-05-08

**Authors:** Georgios Fountoukidis, Piotr Jakuszewski, Frida Jakobsson, Antonis Valachis

**Affiliations:** 1 Department of Oncology, Faculty of Medicine and Health, Örebro University, Örebro, Sweden; 2 Department of Renal Medicine, Faculty of Medicine and Health, Örebro University, Örebro, Sweden

**Keywords:** donor transmitted cancer, graft rejection, immunotherapy, pseudoprogression (PsP), renal failure

## Abstract

We report a case of suspected donor-transmitted squamous cell carcinoma (SCC) in a kidney transplant recipient who received an organ from a deceased donor with no evidence of malignancy at the time of procurement. Approximately one year after transplantation, the recipient developed metastatic SCC with bone and lung involvement. Molecular genotyping was inconclusive regarding donor origin, as tumor cells lacked human leukocyte antigen (HLA) expression. Notably, a second recipient who received the liver from the same donor also developed SCC and subsequently died, raising suspicion of donor transmission. Allograft nephrectomy and complete withdrawal of immunosuppression were initially considered; however, the patient declined due to concerns regarding quality of life associated with dialysis. Systemic chemotherapy was initiated but resulted in disease progression after two months. Treatment was subsequently switched to the checkpoint inhibitor cemiplimab, with concurrent discontinuation of all immunosuppressive therapy except prednisone. Acute graft rejection occurred after two cycles, and the patient resumed dialysis. Subsequent imaging demonstrated findings consistent with pseudoprogression before achieving near-complete remission. The patient completed two years of immunotherapy, after which treatment was discontinued, and remains under surveillance with ongoing near-complete remission. This case highlights the potential role of checkpoint inhibition combined with immunosuppression withdrawal as a therapeutic strategy in in selected patients with donor-transmitted SCC following kidney transplantation.

## Introduction

Donor-transmitted malignancies are rare in organ transplant recipients, with an estimated incidence of approximately 0.2% for cancer transmission (1).

According to international biovigilance frameworks, donor-related malignancies may be classified as donor-derived (arising from donor cells after transplantation) or donor-transmitted (present but undetected at the time of organ procurement) (2).

The most commonly reported donor-transmitted cancers include renal cell carcinoma, lymphoma, melanoma, and Kaposi’s sarcoma, among others (3, 4). However, squamous cell carcinoma (SCC) is less commonly reported. Recipients of donor-transmitted renal carcinoma generally have a more favorable prognosis, with a 5-year overall survival rate of 93% (4). Many of these tumors are identified within the first-year post-transplantation, possibly due to routine imaging during graft function evaluation (4) but not much is known about SCC.

The current case report describes the occurrence of a donor-transmitted SCC in a kidney transplant recipient, where the donor origin of malignancy was suspected in a prior liver transplant recipient. It emphasizes the challenges in managing donor-transmitted cancers, particularly in transplant recipients receiving immunotherapy with checkpoint inhibitors (CPIs). Despite the associated risk of graft rejection, estimated to be around 40% (5), CPIs may still play a potential role in treating such cases. However, there are few case reports in the literature describing the use of CPIs for donor-transmitted cancers (6–9).

## Patient information

The patient, a female in her 70s, with a history of unspecified glomerulonephritis who had been on peritoneal dialysis for 2 years received a kidney transplant from a deceased donor. No history of malignancy was documented in the donor at the time of organ recovery. Another recipient had previously undergone a liver transplant from the same donor, which was complicated by the development of SCC in the liver. The liver transplant recipient ultimately succumbed from the disease. As soon as this was discovered, the patient was offered by the transplantation center allograft nephrectomy and withdrawal of immunosuppressive therapy, but she declined due to concerns about post-dialysis quality of life. Six months before the onset of symptoms, a PET-CT (Positron Emission Tomography - Computed Tomography) scan had been performed and showed no signs of malignancy. However, the patient later presented with new back and hip pain, prompting further investigation. A CT scan revealed a 4 cm area of bone destruction in the left iliac bone, suspicious lesions in L4, and lung nodules.

## Clinical findings and diagnosis

A biopsy of the lesion from left iliac bone confirmed SCC. Clinical examination did not show signs of primary cutaneous SCC. PET-CT showed an expansive process in the graft. Molecular genotyping to determine the origin of malignancy was initially inconclusive, as no Human leukocyte antigens (HLA) expression was detected in the tumor cells. A second molecular test for a Y chromosome, due to the male donor and female recipient, also yielded inconclusive results. The patient’s case was reviewed by a tertiary transplantation center in Sweden, and the possibility of allograft nephrectomy and withdrawal of immunosuppression was discussed, based on reports of spontaneous remission of donor-derived malignancies following immunosuppressive withdrawal (10). However, the patient declined this option because of concerns regarding quality of life on dialysis. Moreover, there were concerns due to the lack of evidence confirming whether the patient’s cancer was donor-transmitted and whether removal of the transplant would be beneficial.

## Treatment and outcome

Considering the lack of confirmation regarding the origin of SCC and the patient’s preference, we chose to proceed with chemotherapy consisting of carboplatin AUC 5 and paclitaxel 175mg/m^2^ every three weeks. Immunosuppressive therapy continued (tacrolimus 2 + 1.5 mg and everolimus 0.75 mg twice daily), with a reduction of prednisone to 2.5 mg once daily. After three cycles of chemotherapy, the patient showed partial tumor regression in the transplanted kidney and lung metastases but progression of bone metastases, with new lesions in the right iliac bone. The patient received radiotherapy (8 Gy in one fraction) against two bone lesions in left iliac bone and acetabulum. An additional CT scan few weeks after the administration of radiotherapy confirmed progressive disease.

After discussion in a multidisciplinary team meeting with the Transplantation Center, it was decided to initiate CPI with cemiplimab in a standard dose of 350 mg every three weeks and to withdraw all immunosuppressive therapy other than prednisone at a dose of 20 mg once daily in order to minimize immunosuppression while allowing antitumor immune activation. The patient received an additional radiation dose (8 Gy in one fraction) to the left hip area for pain relief. After two cycles of cemiplimab, the patient developed graft rejection with acute renal failure, generalized edema with increased body weight, and diarrhea. A CT scan revealed bilateral pulmonary ground-glass opacities, initially suspected to be infection or pneumonitis. The patient was treated with antibiotics (cefotaxim 1g x 2) against suspected lung infection, high-dose corticosteroids (methylprednisolone 2mg/kg intravenously), and oral vancomycin for Clostridioides *difficile* infection that was diagnosed in parallel. She gradually responded to treatment, and the corticosteroids were tapered. The lung ground-glass opacities were attributed to fluid overload. The patient started dialysis and discharged after one month.

## Treatment response and follow-up status

A follow-up PET-CT scan performed one month after discharge showed signs of disease progression, with more pronounced bone lesions and regional lymph nodes demonstrating increased FDG uptake, while the lung metastases remained in remission. Given the potential for pseudoprogression, it was decided to continue treatment with the CPI and schedule a repeat PET-CT scan after eight weeks. The subsequent scan revealed near-complete metabolic remission, with only minimal FDG uptake in the left iliac bone, confirming the initial suspicion of pseudoprogression.

The patient completed a total of two years of cemiplimab treatment, after which treatment was discontinued, and is currently under regular clinical and radiological follow-up. The patient remains in near-complete remission, with no significant treatment-related side effects. However, she continues to require hemodialysis, which has a substantial impact on her quality of life.

**Figure 1:**
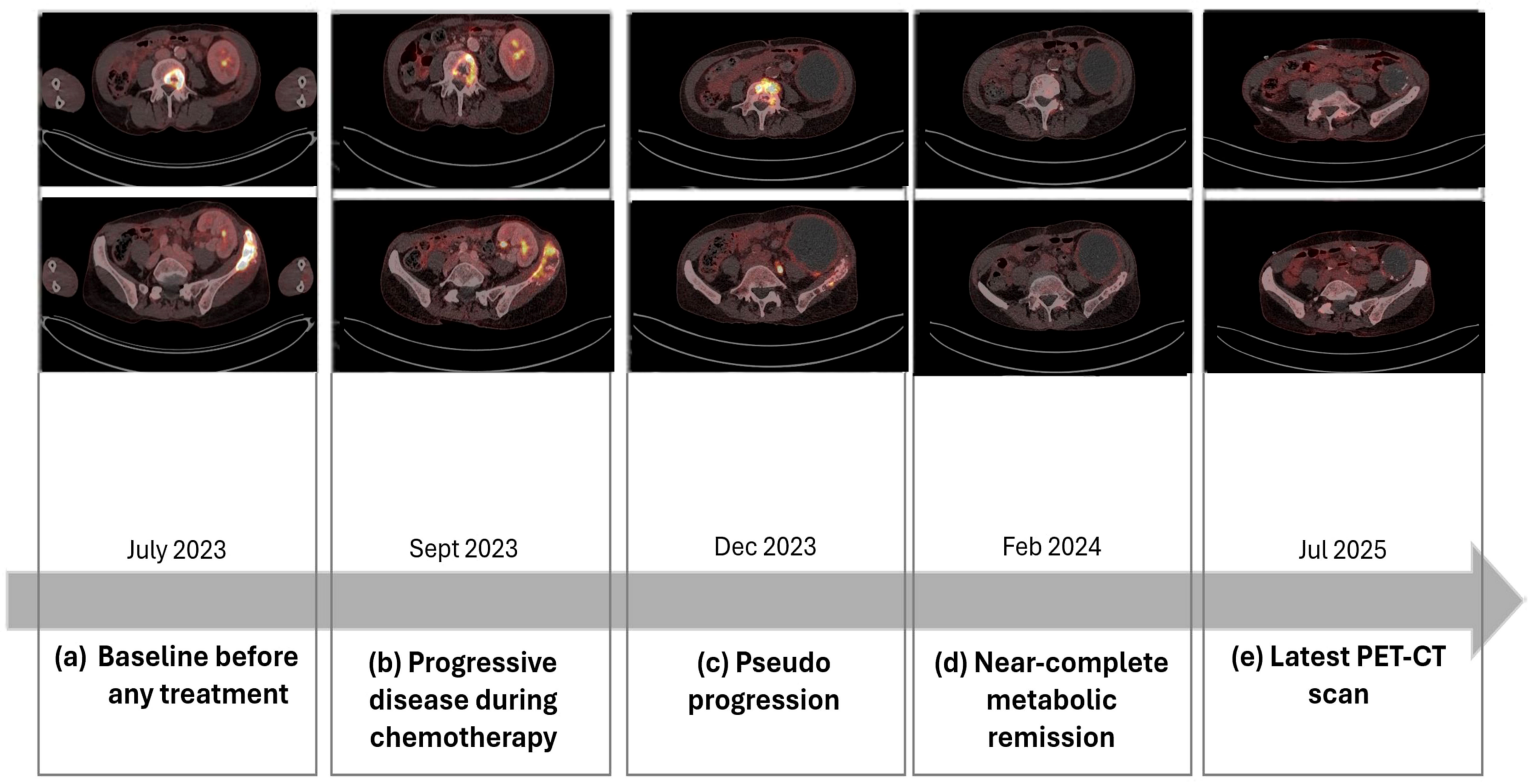
Timeline of clinical course from cancer diagnosis to latest follow-up.

**Figure 2:**
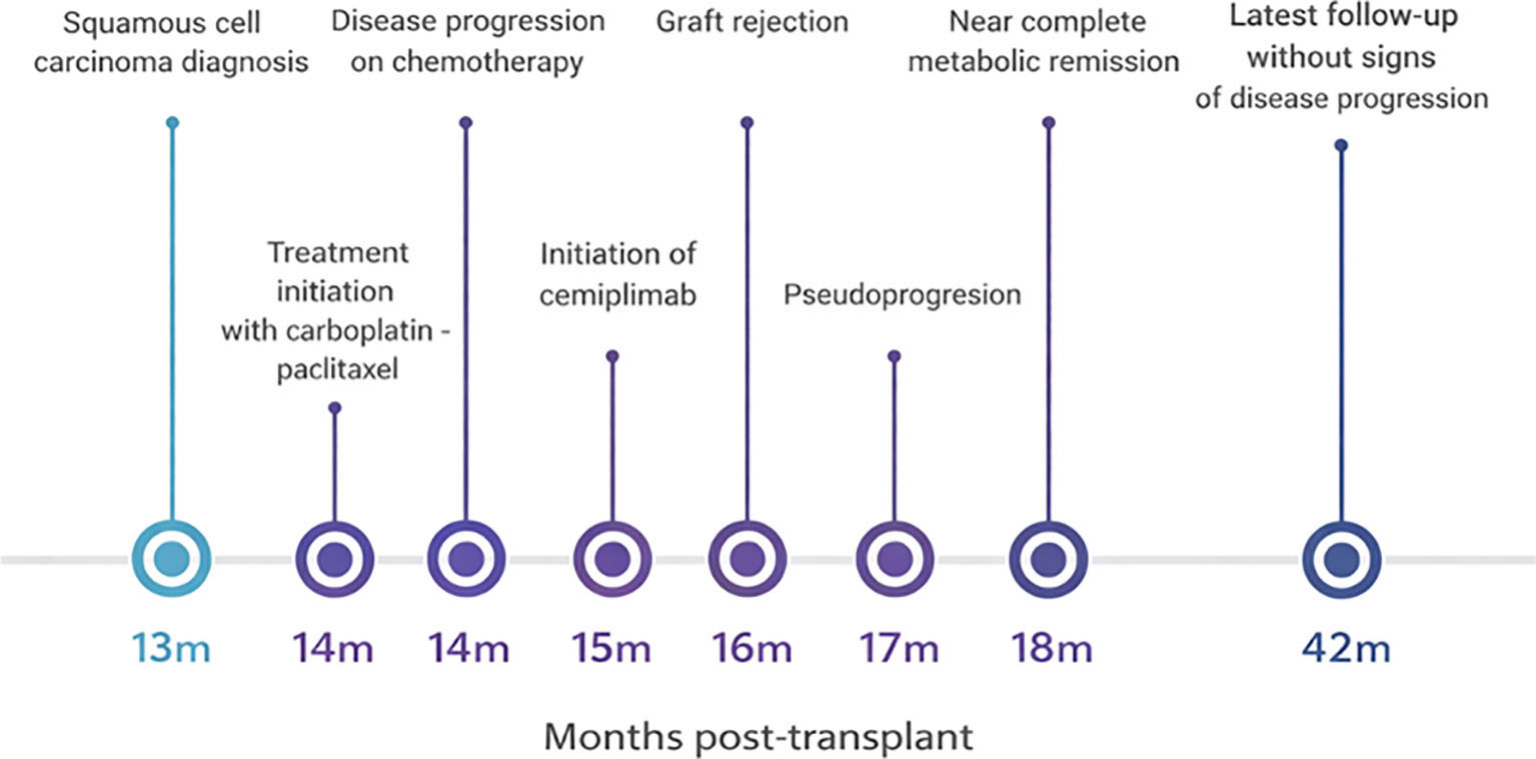
Changes in PET-CT findings over time, from pre-treatment baseline to latest scan, showing consistent near-complete metabolic remission.

A timeline of clinical events from the initial diagnosis of malignancy to the latest follow-up is shown in **Figure 1**. **Figure 2** illustrates the changes of PET-CT findings throughout the course of treatment.

## Discussion

Donor-transmitted malignancies in transplant recipients are rare and challenging to manage. This case highlights the potential role of immunotherapy, particularly CPIs, in treating donor-transmitted SCC. It remains difficult to determine whether tumor control could have been achieved solely through withdrawal of immunosuppression, without the addition of immunotherapy. While the combination of immunosuppressive withdrawal and immunotherapy led to significant disease regression, it ultimately led to kidney graft rejection—highlighting the need for careful management of both oncologic control and transplant function. As a result, identifying the balance between cancer treatment and graft preservation must be tailored to each individual case.

Definitive molecular confirmation of donor origin was not available in this case. Although short tandem repeat (STR) genotyping can establish donor–recipient tumor origin through genomic comparison (11), these analyses were not accessible to the treating oncology team. Similarly, comparative molecular analysis between tumors from the kidney and liver recipients was not available. Nevertheless, the occurrence of histologically similar SCC in two recipients from the same donor, within a comparable timeframe and in the absence of prior malignancy, strongly supports a donor-related event. The likelihood of two independent primary tumors with overlapping morphology is considerably lower than that of transmission from a common donor source. According to international biovigilance classification frameworks (2) distinguishing donor-derived from donor-transmitted malignancies, this case is most consistent with a probable donor-transmitted malignancy. Future cases would benefit from early multidisciplinary coordination to enable timely genomic characterization and strengthen diagnostic certainty.

There are only a few cases in the literature describing the use of CPIs in patients with donor-transmitted cancer (6–9). In all four cases, the transplant graft was surgically removed either as a part of treatment strategy against cancer or due to graft rejection. Boyle et al. (6) reported a case of donor-derived melanoma treated initially with nephrectomy, followed by BRAF/MEK inhibitors, and ultimately with nivolumab, achieving a good response. Amara et al. (7) described a renal and pancreas transplant recipient diagnosed with donor-derived renal cell carcinoma. The patient underwent nephrectomy and, several years later, developed metastatic disease. Treatment included cessation of immunosuppressive therapy and administration of nivolumab. Although the patient responded to the CPI, an urgent transplant pancreatectomy was required due to graft rejection. Kommer et al. (8) presented a case of a kidney transplant recipient with donor-transmitted melanoma. The patient was initially treated with BRAF and MEK inhibitors combined with discontinuation of immunosuppressive therapy, which led to urgent nephrectomy. Due to disease progression, treatment with ipilimumab and nivolumab was initiated, followed by a rechallenge with BRAF and MEK inhibitors due to further progression. In the case presented by Singh et al. (9), a patient with donor-transmitted metastatic melanoma was first managed by discontinuing immunosuppressive medications and undergoing nephrectomy due to rejection. The patient was subsequently treated with BRAF/MEK inhibitors and eventually received ipilimumab, achieving complete remission.

In 2024, a Phase 1 study suggested that mTOR inhibitors combined with corticosteroids may offer a favorable immunosuppressive regimen for kidney transplant recipients receiving CPIs for advanced cutaneous SCC, with no reported cases of graft rejection (12). In contrast, a prospective clinical study indicated that the combination of tacrolimus and prednisone might provide insufficient allograft protection and could compromise immune-mediated tumor regression when nivolumab (with or without ipilimumab) is used (13) Management of the kidney allograft represented a major therapeutic challenge. High-dose corticosteroids were administered for suspected immune-related pneumonitis rather than as targeted anti-rejection therapy. Despite treatment, graft function progressively declined, ultimately resulting in dialysis dependence. In the context of clinical deterioration and the need to preserve effective antitumor immune activation, oncologic disease control was prioritized over escalation of immunosuppression. Invasive diagnostic procedures, including graft biopsy, were deferred as they were unlikely to alter immediate management. Although donor-specific antibody testing and emerging biomarkers such as donor-derived cell-free DNA may provide additional insight into rejection mechanisms (13), these data were not available. Transplantectomy was discussed but not pursued following multidisciplinary evaluation. This case underscores the complexity of balancing immune checkpoint inhibitor efficacy against the risk of allograft rejection in transplant recipients, where competing priorities often necessitate individualized, risk-adapted decision-making. Another important consideration is whether patients with donor-transmitted cancer who achieve remission could be eligible for a new transplantation. Although data are limited, several case reports suggest that this approach may be feasible in select cases (7). In the present case, the transplantation center recommended prolonged oncologic follow-up, potentially up to five years without disease recurrence, before reconsidering eligibility. Patient age and evolving clinical evidence will also influence future decisions.

## Conclusion

This case highlights the potential of immunotherapy to treat donor-transmitted SCC in kidney transplant recipients. Although the treatment achieved near-complete remission of cancer, the patient ultimately experienced graft rejection, underscoring the importance of carefully managing both cancer therapy and transplant function. Further studies are needed to better understand the long-term outcomes of immunotherapy in organ transplant recipients with donor-transmitted cancers and to develop optimal strategies for balancing effective cancer treatment with graft preservation.

## Data Availability

The original contributions presented in the study are included in the article/supplementary material. Further inquiries can be directed to the corresponding author.
